# Differential effects of sex on amyloid lowering by Aducanumab and IgG in 5xFAD mice

**DOI:** 10.1002/alz70859_105982

**Published:** 2025-12-26

**Authors:** Kathryn A Haynes, Zackery A Cope, Sean‐Paul Gerard Williams, Umesh Nepali, Gabriela Little, Suzanne Doolen, Emma H. Doud, Lais da Silva, Sara K Quinney, Kierra Eldridge, Charlie P Burton, Scott A Persohn, Ravi S Pandey, Gregory W Carter, Michael Sasner, Bruce T. Lamb, Adrian L Oblak, Paul R Territo, Stacey J Sukoff Rizzo

**Affiliations:** ^1^ University of Pittsburgh School of Medicine, Pittsburgh, PA USA; ^2^ Indiana University School of Medicine, Indianapolis, IN USA; ^3^ The Jackson Laboratory, Bar Harbor, ME USA

## Abstract

**Background:**

While there is ongoing support to position amyloid‐targeting antibody‐based therapeutics as standard‐of‐care treatments for Alzheimer’s disease (AD), their benefits and limitations are debated. Despite significant reductions in amyloid with treatment, the disease continues to progress, and clinical trial data have now clearly demonstrated differential effects of sex. Given that females have a higher risk of AD, it is prudent to understand and predict beneficial effects and limitations in both sexes. The present studies aimed to characterize a murinized chimeric Aducanumab (^ch^Aducanumab) through the MODEL‐AD Preclinical Testing Core (PTC) Drug Screening Pipeline. We evaluated the pharmacokinetic (PK), pharmacodynamic (PD), and behavioral effects of chronic administration to 5XFAD mice in comparison to murine IgG2a kappa control antibody (IgG) and saline.

**Methods:**

5XFAD mice with significant plaque accumulation were chronically dosed IP once weekly ^ch^Aducanumab and compared to IgG and saline controls. Brain and plasma levels of ^ch^Aducanumab were analyzed using an attomolar sensitive, targeted mass spectrometry parallel reaction monitoring assay. Pre‐ and post‐treatment plasma Aβ, terminal soluble and insoluble brain Aβ, and in vivo 18F‐AV‐45 and 18F‐FDG PET were quantified and cross‐referenced for the presence of anti‐drug antibodies. Behaviors and cognitive function were tested in a behavioral battery.

**Results:**

Both ^ch^Aducanumab and IgG were effective in attenuating plasma and brain Aβ with more robust effects on Aβ lowering in males than in females, and dose‐dependent levels of ^ch^Aducanumab were detectable in plasma and brain homogenate. Reduction of Aβ plaques did not correspond to significant improvements in performance on cognitive tasks or healthspan measures. The variability in PD responses within ^ch^Aducanumab treatment groups was cross‐referenced to individual data for the presence of anti‐drug antibodies.

**Conclusions:**

Sex‐dependent differential clearance of amyloid plaques with monoclonal antibody treatment has been observed translationally across species. The interaction with IgG may contribute to both the amyloid‐lowering effects and possibly to the reported adverse events. Results from this study will inform future MODEL‐AD PTC evaluations of novel therapeutics, particularly those combined with or following anti‐Aβ immunotherapies once amyloid is lowered and the disease continues to progress, and importantly to identify and predict differential effects of sex.

